# Diagnosis of cytomegalovirus pneumonia by quantitative polymerase chain reaction using bronchial washing fluid from patients with hematologic malignancies

**DOI:** 10.18632/oncotarget.14504

**Published:** 2017-01-04

**Authors:** Hwa Young Lee, Chin Kook Rhee, Joon Young Choi, Hea Yon Lee, Jong Wook Lee, Dong Gun Lee

**Affiliations:** ^1^ Department of Internal Medicine, Division of Pulmonary and Critical Care Medicine, The Catholic University of Korea, Seoul, Korea; ^2^ Department of Internal Medicine, Division of Hematology, The Catholic University of Korea, Seoul, Korea; ^3^ The Catholic Blood and Marrow Transplantation Center, The Catholic University of Korea, Seoul, Korea; ^4^ Department of Internal Medicine, Division of Infectious Diseases, The Catholic University of Korea, Seoul, Korea; ^5^ Vaccine Bio Research Institute, The Catholic University of Korea, Seoul, Korea

**Keywords:** cytomegalovirus, pneumonia, real-time polymerase chain reaction, hematologic neoplasms

## Abstract

**Background:**

The incidence of cytomegalovirus (CMV) pneumonia is increasing in patients diagnosed with hematologic malignancies. The utility of CMV-DNA viral load measurement has not been standardized, and viral cut-off values have not been established. This study was designed to investigate the utility of CMV quantitative real-time PCR (qRT-PCR) using bronchial washing fluid.

**Methods:**

We retrospectively reviewed the microbiologic and pathologic results of bronchial washing fluid and biopsy specimens in addition to the patients' clinical characteristics.

**Results:**

A total of 565 CMV qRT-PCR assays were performed using bronchial washing fluid from patients with hematologic malignancies. Among them, 101 were positive for CMV by qRT-PCR; of these, 24 were diagnosed with CMV pneumonia and 70 with CMV infection, and 7 were excluded due to a diagnosis of invasive pulmonary aspergillosis rather than viral pneumonia. The median CMV load determined by qPCR was 1.8 × 10^5^ copies/mL (3.6 10^3^-1.5 × 10^8^) in CMV pneumonia patients and 3.0 × 10^3^ copies/mL (5.0 × 10^2^-1.1 × 10^5^) in those diagnosed with CMV infection (*P* < 0.01). Using the ROC curve, the optimal inflection points were 18,900 copies/mL (137,970 IU/mL) in post-bone marrow transplantation (BMT) patients, 316,415 copies/mL (2,309,825 IU/mL) in no-BMT patients and 28,774 copies/mL (210,054 IU/mL) in all patients.

**Conclusions:**

The CMV titers in bronchial washing fluid determined by qRT-PCR differed significantly between patients diagnosed with CMV pneumonia and those with CMV infection. The viral cut-off values in bronchial washing fluid were suggested for the diagnosis of CMV pneumonia, which were different depending on the BMT status.

## INTRODUCTION

Cytomegalovirus (CMV) is a common pathogen that causes syndromes ranging from asymptomatic viremia to end-organ diseases [1, 2]. CMV infection can result in high morbidity and mortality rates, especially in immunocompromised patients with cell-mediated immunodeficiencies. The incidence of CMV pneumonia in patients diagnosed with hematologic malignancies has been reported to range from 2.3% to 16%, with an overall mortality rate of 57%, which is increasing [3]. There are some literatures describing non-transplant patients with the risks of CMV pneumonia such as chronic lymphocytic leukemia or lymphoma, use of rituximab, systemic steroid, alemtuzumab or busulfan [4–8]. Development of immunosuppressive agents and increased use of CMV polymerase chain reaction (PCR) in bronchoalveolar lavage (BAL) fluid or serum would have increased the incidence of CMV pneumonia in non-transplant patients with hematologic malignancies. However, diagnostic methods for CMV pneumonia have not undergone marked development in the past few decades [9]. Definitive diagnosis of CMV pneumonia is determined based on a combination of symptoms and signs of pulmonary disease and detection of CMV in BAL fluid or lung tissue samples by virus isolation, histopathologic testing, immunohistochemical analysis or in *in situ* hybridization [1, 2].

PCR has been used to detect CMV since the early 1990s and is the most sensitive method of detecting CMV. Quantitative real-time PCR (qRT-PCR) can be used to quantify viral loads in blood and BAL fluid. The viral load in blood is a good predictor of CMV disease and is used for pre-emptive therapy after bone marrow transplantation (BMT) [10–12]. However, the viral load of BAL fluid determined by qRT-PCR has a low specificity and positive predictive value despite its high sensitivity and negative predictive value [2].

Moreover, asymptomatic lung viral infections have been reported in patients with hematologic malignancies and in immunocompetent hosts without any evidence of acute CMV disease [13, 14]. Thus, it remains very difficult to distinguish CMV infection from CMV end-organ disease. The question of whether viral titers are higher in patients with CMV pneumonia than in those exhibiting asymptomatic pulmonary shedding remains controversial. Methods of measuring CMV DNA viral loads have not been standardized, and no viral cut-off value distinguishing CMV infection from CMV pneumonia has been established [1, 9].

As far as we are aware, there are no defined viral cut-off values from samples other than whole blood to diagnose CMV disease in patients with hematological malignancies; only CMV DNAemia could be diagnosed with CMV real-time PCR. In lung transplant recipients, CMV culture of BAL fluid has been sensitive but less useful compared with histological assessment of transbronchial biopsies [15, 16]. Regarding the clinical utility of CMV load in BAL fluid, one clinical study in 2005 reported that >500,000 copies/mL (mean, 1,638,450 copies/mL) had 100% sensitivity and specificity compared with positive lung biopsies using a quantitative hybrid capture assay [17]. However, other than this report, studies aiming to identify CMV qRT-PCR cut-off values in BAL fluid are lacking.

This study aimed to evaluate the diagnosis of CMV pneumonia in patients with hematologic malignancies using CMV qRT-PCR and to define the viral load cut-off values that enable discrimination of CMV pneumonia from CMV infection using bronchial washing fluid specimens.

## MATERIALS AND METHODS

### Data collection and patients

We identified 565 adult patients over 15 years of age with hematologic malignancies who underwent bronchoscopy to isolate the causative pathogen of pneumonia from March 2008 to June 2014 at Seoul St. Mary's Hospital (Seoul, Republic of Korea), where over 450 BMTs are performed annually. The clinical diagnostic criteria of pneumonia included a new infiltrate on chest radiograph, clinical signs of lower respiratory tract infection and/or fever. The microbiological findings from bronchial washing fluids in these patients were analyzed retrospectively, and additional assays such as blood CMV qRT-PCR and lung biopsies were also reviewed. Data regarding the following clinical characteristics of the patients were collected: age, sex, hematologic diagnosis, prior hematologic treatment and transplantation characteristics.

This study was approved by the Institutional Review Board of Seoul St. Mary's Hospital, which waived the requirement for informed consent (No. KC15RISI0153).

### Fiber-optic bronchoscopy procedure

Bronchoscopy was performed after identification of pulmonary infiltration on chest computed tomography (CT). All bronchoscopic examinations were performed using a flexible bronchoscope (BF-1T60t, Olympus, Tokyo, Japan) by an experienced bronchoscopist. Bronchial washing was conducted after the bronchoscope was wedged into a segmental bronchus consistent with the newly developed infiltrate detected by chest CT, and 10 mL normal saline were repeatedly instilled until at least 20 mL of the aspirate had been collected in the trap bottle.

### Microbiologic assays

Direct examination and culture for bacteria, fungi and viruses were performed on the bronchoscopic washing fluid specimens. For detection of bacteria, mycobacteria and fungi, Gram, Ziehl–Neelsen, Periodic-acid Schiff, and Gomori methenamine silver staining analyses of tissue were performed, followed by culture. For the detection of respiratory viruses, a multiplex qPCR assay was performed for influenza viruses A and B; parainfluenza viruses 1, 2 and 3; respiratory syncytial virus; adenovirus; metapneumovirus; rhinovirus A, B, and C; coronavirus; and bocavirus (AdvanSure RV Real-time PCR kit, LG Life Sciences, Seoul, Korea). Polymerase chain reaction assays were also used to detect *Pneumocystis jirovecii* and mycobacteria [18, 19].

CMV was detected by qRT-PCR of CMV DNA, shell vial culture, and immunohistochemical (IHC) staining for CMV in bronchial washing fluid. Moreover, hematoxylin–eosin and IHC stainings for CMV in transbronchial lung biopsies were reviewed by an experienced lung pathologist. For qRT-PCR of CMV, DNA was extracted from 200 μL whole blood or bronchial washing fluid using a QIAamp DNA Blood Kit (Qiagen, Valencia, CA, USA) in accordance with the manufacturer's instructions [20]. RT-PCR for CMV DNA was performed using the ExiCycler™ 96 instrument (Bioneer Corporation, Daejeon, Korea) and AccuPower^®^ CMV Quantitative PCR Kit (Bioneer). To establish the limit of detection (LoD), we amplified control samples (1,000, 333, 111, 37, 12, and 4 copies/mL) twice daily for 10 days (two sets, a total of 40 samples) and performed probit analysis. The LoD was 380 copies/mL. For the standardization of the results, the WHO (World Health Organization) International Standard for human CMV for nucleic acid amplification techniques (National Institute for Biological Standards and Control [NIBSC] code: 09/162) was used. The WHO international standard was diluted to 312,500, 31,250, 3,125, and 312.5 IU/mL, and 12 replicates at each concentration were run on 4 separate days. The data collected in copies/mL were compared to the expected IU/mL. A conversion factor was calculated by taking the mean ratio of IU/mL to copies/mL for all data points. One copy of CMV DNA using the AccuPower^®^ CMV Quantitative PCR test was equivalent to 7.3 International Unit (IU).

### Definition of CMV pneumonia

CMV pneumonia was suspected in patients who presented with signs and symptoms of pneumonia and chest CT findings compatible with viral pneumonia. CMV pneumonia was defined according to established criteria [1]. CMV diagnosis was categorized as proven, probable, possible or indeterminate by an experienced specialist (Lee DG) from the Division of Infectious Diseases according to a retrospective review of individual chart and chest CT findings (Table 1) [21–23]. Patients in whom co-pathogens (such as *Aspergillus* spp.) were detected in bronchial washing fluid and who had radiologic signs typical of invasive pulmonary aspergillosis (IPA) were excluded.

**Table 1:** Definition of CMV pneumonia

Classification	
Proven	Positive CMV virus culture in bronchial washing fluid or the presence of intranuclear inclusion body or detection of CMV using immunohistochemical staining in a lung biopsy specimen
Probable	Presence of intranuclear inclusion body or detection of CMV using immunohistochemical staining in a cytologic specimen of bronchial washing fluid
Possible	Not classified as proven or probable
Indeterminate	Not classified as proven or probable and common respiratory virus other than CMV was isolated

### Statistical analyses

All statistical analyses were performed using SPSS software (ver. 18.0.0 for Windows; SPSS, Inc., Chicago, IL, USA). All results are expressed as means ± SEM for continuous variables and proportions for categorical variables. CMV qRT-PCR results are presented as means, medians and ranges. Differences in CMV PCR titers between patients diagnosed with and without CMV pneumonia were analyzed using the Mann–Whitney U-test. All tests were two-sided, and a *P* value < 0.05 was considered to indicate statistical significance. To identify the optimal viral load cut-off value for differentiating patients diagnosed with CMV pneumonia from those with CMV infection, operating characteristics (sensitivity and specificity) were calculated for each cut-off value, and a receiver operating characteristics (ROC) curve was plotted to determine the most accurate cut-off point.

## RESULTS

### Patient characteristics

**Figure 1:**
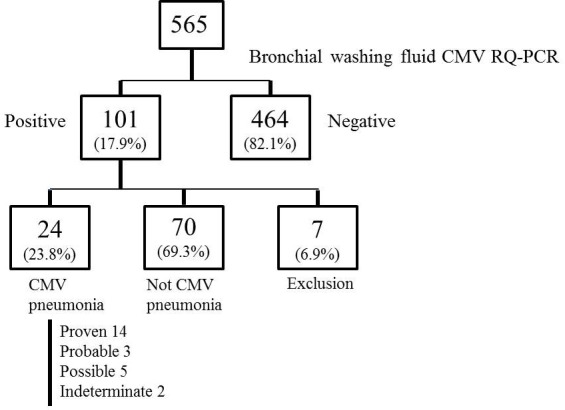
Flow chart of patients with hematologic malignancies who underwent bronchoscopy during the study period

Bronchoscopy was performed a mean of 2.54 ± 2.9 days after identification of new pulmonary infiltrates suggestive of pneumonia. Among the initially identified 565 patients, 464 (82.1%) were negative for CMV or exhibited <380 copies/mL (2,470 IU/mL) CMV DNA by qRT-PCR, while 101 (17.9%) harbored > 380 copies/mL. According to the consensus criteria, 24 (23.8%) patients were diagnosed with CMV pneumonia, 70 (69.3%) exhibited findings inconsistent with CMV pneumonia, and 7 (6.9%) were excluded due to suspicion of proven IPA. Of the 24 patients diagnosed with CMV pneumonia, 14 (58.3%) were classified as proven CMV pneumonia, 5 (20.8%) possible, 3 (12.5%) probable and 2 (8.3%) indeterminate (Figure 1, Supplementary Figure 1). Nine cases were classified as proven based on positive CMV culture of bronchial washing fluid and five of lung biopsy specimens. Two indeterminate cases were classified as coinfections with other respiratory viruses: coronavirus and rhinovirus.

Baseline characteristics of the patients and prior treatments are shown in Table 2. The mean age was 48 ± 3.0 years, and 75% of the patients were male. The most common underlying hematologic diseases were acute myeloid leukemia (25.0%), hemophagocytic lymphohistiocytosis (16.7%), non-Hodgkin's lymphoma (12.5%) and aplastic anemia (12.5%). Sixteen (66.7%) patients were using immunosuppressant agents at the time of diagnosis of CMV pneumonia, and 12 (46.2%) had received systemic chemotherapy—including alemtuzumab in 2 and steroid pulse therapy (methylprednisolone >1 mg/kg/day) in 3 (12.5%)—due to aggravation of GVHD in the last 30 days. All 24 patients diagnosed with CMV pneumonia were treated with antiviral agents, which resulted in aggravation of pneumonia in 14 (58.3%) and improvement in 10 (41.7%) patients. During the mean 122 days of follow up, 15 (62.5%) patients died, and the 28-day mortality rate was 45.8%. Death was due to CMV pneumonia in 14 (58.3%) patients. Among the total 24 patients, 15 patients (62.5%) were co-infected. Seven (46.7%) patients were co-infected by 4 bacteria (*Staphylococcus aureus, Pseudomonas aeruginosa, Stenophomonas maltophilia* and Mycoplasma), 3 (20.0%) by 3 viruses (coronavirus, parainfluenza virus and rhinovirus), 3 (20.0%) by 2 fungi (*Pneumocystis jirovecii* and *Aspergillus niger*) and 2 (13.3%) by Mycobacterium species (*Mycobacterium tuberculosis* and non-tuberculous mycobacterium).

**Table 2:** Baseline clinical characteristics of the patients (*n* = 24)

	Mean ± SEM or No (%)
Age (yr)	48 ± 3.0
Male, n (%)	18 (75.0)
Underlying hematologic disease, n (%) Acute myeloid leukemia Hemophagocytic lymphohistiocytosis Non-Hodgkin's lymphoma Aplastic anemia Acute lymphoblastic leukemia Myelodysplastic syndrome Multiple myeloma Chronic myelogenous leukemia Chronic lymphocytic leukemia Primary myelofibrosis	6 (25.0) 4 (16.7) 3 (12.5) 3 (12.5) 2 (8.3) 2 (8.3) 1 (4.2) 1 (4.2) 1 (4.2) 1 (4.2)
Prior treatment, n (%) Bone Marrow transplantation Use of immunosuppressant agent Systemic chemotherapy in last 30 days Alemtuzumab chemotherapy	16 (66.7) 16 (66.7) 12 (46.2) 2 (8.3)
Steroid pulse therapy in last 30 days History of preemptive CMV therapy, n (%)	3 (12.5) 4 (16.7)
Prognosis of pneumonia, n (%) Improved Aggravated Follow up periods (days)	10 (41.7) 14 (58.3) 122.0 ± 65.0
Death, n (%) Survived Before 28 days of diagnosis After 28 days of diagnosis Death due to the pneumonia, n (%)	15 (62.5) 9 (37.5) 11 (45.8) 4 (16.7) 14 (58.3)

SEM : Standard Error of the Mean

CMV : cytomegalovirus

Steroid pulse therapy : Methylprednisolone > 1 mg/kg/day

Table 3 shows the transplantation characteristics of the patients diagnosed with CMV pneumonia. Of the 24 diagnosed patients, 16 had undergone BMT. The mean time to diagnosis of CMV pneumonia after BMT was 167.7 ± 60.9 days; however, the majority of the patients (n=11, 68.8%) were diagnosed <100 days after BMT. Four patients had histories of pre-emptive CMV therapy from the time of undergoing BMT to diagnosis of CMV pneumonia. In all of these patients, CMV pneumonia was diagnosed during the late period, 100 days (range 215–1006 days, data not shown) after BMT. The most frequent donors were siblings, and two patients had undergone auto-transplantations.

**Table 3:** Transplantation characteristics of the patients diagnosed with CMV pneumonia (*n* = 16)

	Mean ± SEM or No (%)
Donor type, n (%) Sibling Unrelated Familial missmatched transplantation Cord Autologous	5 (31.3) 4 (25.0) 4 (25.0) 1 (6.3) 2 (12.5)
Source of graft, n (%) Bone marrow Peripheral blood stem cell Cord blood stem cell	2 (12.5) 13 (81.3) 1 (6.3)
Risk of CMV disease, n (%) High risk† Low risk‡	10 (62.5) 6 (37.5)
Time since BMT (days) Before 100 days after BMT, n (%)	167.7 ± 60.9 11 (68.8)

SEM : Standard Error of the Mean

CMV : cytomegalovirus

BMT : Bone marrow transplantation

† Patients who had unrelated donors, mismatched related donors, and related donors with acute graft-versus-host disease (GVHD) of grades II–IV or severe chronic GVHD

‡ Patients who had related donors with acute GVHD of grade I or without acute/chronic GVHD.

### qRT-PCR of bronchial washing fluid

**Figure 2:**
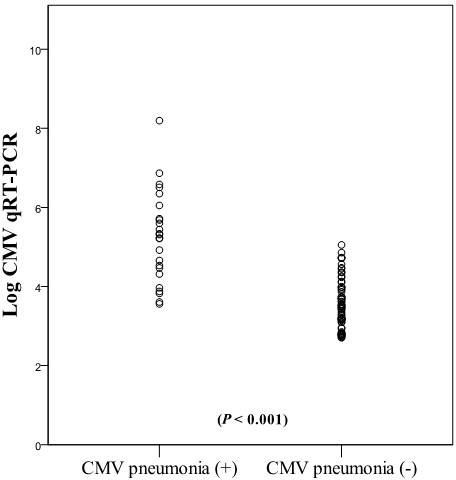
Distribution of CMV viral load in bronchial washing fluid The median log_10_ (CMV qRT-PCR copies/mL) values between the patients diagnosed CMV pneumonia and those who were not diagnosed CMV pneumonia were different significantly (*P* < 0.001).

Figure 2 shows the distribution of CMV qRT-PCR viral load in bronchial washing fluid. The median log_10_ (CMV qRT-PCR copies/mL) values were 5.3 (range, 3.56–8.19) in patients diagnosed with CMV pneumonia (n=24) and 3.4 (range 2.7-5.05) in those who were not diagnosed with CMV pneumonia (n=70); this difference was significant (*P* < 0.001). Table 4 shows the qRT-PCR results of patients with (n = 24) versus without a diagnosis of CMV pneumonia (n = 70). The median CMV loads in bronchial washing fluid were significantly different between the two groups (with vs. without CMV pneumonia: 1.8 × 10^5^ copies/mL vs. 3.0 × 10^3^ copies/mL; Mann–Whitney U-test, *P* < 0.001). The median CMV loads in blood were also significantly different between the two groups (3.3 × 10^4^ copies/mL vs. 5.4 × 10^3^ copies/mL, respectively; Mann–Whitney U-test, *P* = 0.006). Patients diagnosed with CMV pneumonia had six-fold higher median blood CMV loads than those who were not diagnosed with CMV pneumonia.

**Table 4:** Comparison of quantitative polymerase chain reaction results between patients diagnosed with CMV pneumonia or not

	CMV pneumonia (*n* = 24)	Not CMV pneumonia (*n* = 70)	*P*
Bronchial washing fluid (copies/ml) Mean Median Minimum - Maximum	7,378,508.6 187,224.5 3,642-156,666,945	10,899.2 3,055 506-113,000	< 0.001
Blood (copies/ml) Mean Median Minimum - Maximum	683,659.1 33,839.5 882-5,570,000	20,915.4 5,486.5 689-280,870	0.006

### Determination of CMV DNA cut-off values

**Figure 3:**
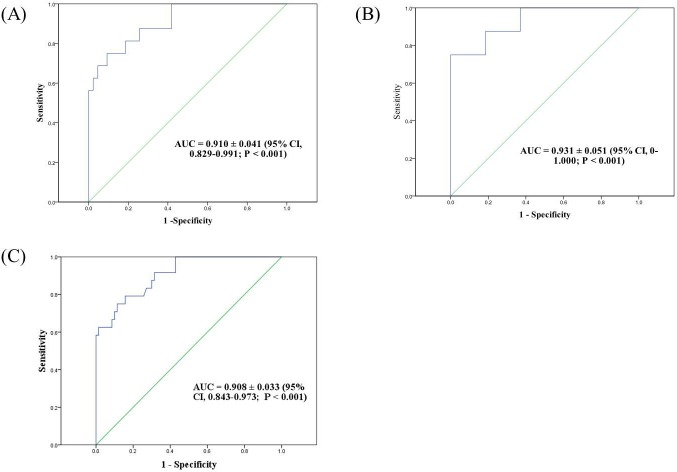
Receiver-operator characteristics (ROC) curve of patients who were diagnosed versus not diagnosed with CMV pneumonia but with a CMV load >380 copies/mL (2,470 IU/Ml) by qRT-PCR **A**. Post-bone marrow transplantation (BMT) patients; viral cut-off was 18,900 copies/mL **B**. no-BMT patients; viral cut-off was 316,415 copies/mL. **C**. All patients; viral cut-off was 28,774 copies/mL.

A receiver operating characteristics (ROC) curve was plotted to identify the optimal cut-off values of CMV load in bronchial washing fluid for a diagnosis of CMV pneumonia. Among 94 patients analyzed, 35 were of no-BMT status and 59 were post-BMT status. Sixteen of the 59 post-BMT patients were diagnosed with CMV pneumonia (Supplementary Figure 1). The ROC curve showed that the optimal inflection point in post-BMT patients was 18,900 copies/mL (137,970 IU/mL) (AUC 0.91 ± 0.041, *P* < 0.001, sensitivity : 81.3%, specificity : 81.4%) (Figure 3A). Among the 35 no-BMT patients, 8 had CMV pneumonia. The viral cut-off level was 316,415 copies/mL (2,309,825 IU/mL) (AUC 0.93 ± 0.051, *P* < 0.001, sensitivity : 100%, specificity 63%) (Figure 3B). Based on the qRT-PCR results of all patients who were diagnosed (n=24) versus not diagnosed with CMV pneumonia but with a CMV load >380 copies/mL (n = 70), the area under the curve (AUC) value was 0.908 ± 0.033 (95% confidence interval (CI), 0.843–0.973; *P* < 0.001), and the optimal inflection point was 28,774 copies/mL (210,054 IU/mL) (sensitivity 75%; specificity 88.6%) (Figure 3C).

## DISCUSSION

This study demonstrated that CMV loads in bronchial washing fluid determined by qRT-PCR could indicate lung involvement in CMV disease. Patients diagnosed with CMV pneumonia had significantly higher qRT-PCR CMV titers than did those with findings inconsistent with CMV pneumonia, both in bronchial washing fluid (61-fold higher) and whole blood (6-fold higher). Moreover, using the ROC curve, a cut-off value of 28,774 copies/ml (sensitivity 75%; specificity 88.6%) CMV DNA in bronchial washing fluid was correlated with CMV pneumonia. As the prevalence of CMV pneumonia in our BMT center is 0.85% - 0.86% [13], the positive and negative predictive values were 5.3% and 99.8% respectively. Our data is valuable in that for the first time we have provided optimal cut point of CMV DNA in patients with hematologic malignancies. This result will help clinicians to diagnose CMV pneumonia more easily in patients with hematologic malignancy. Although pathologic confirmation is gold standard for diagnosis, lung biopsy usually carries high risk of bleeding in patients with hematologic malignancies. Our data suggests that non-invasive bronchial washing procedure may replace invasive lung biopsy in the diagnosis of CMV pneumonia in these patients. Also our cut off points of CMV qRT-PCR could be supplementary information for the early detection of CMV in pulmonary infiltrates of patients with hematologic malignancies.

Detection of CMV in BAL fluid by PCR in BMT recipients was evaluated in the 1990s. Cathomas et al. reported that among 75 patients, 7 (9.3%) had CMV pneumonia and 6 (8%) had CMV infection without pneumonia; a PCR assay showed 100% sensitivity [2]. The relatively low specificity (93.5%) and positive predictive value (58.3%) were compensated for by the additional performance of CMV immunostaining, which resulted in a 100% positive predictive value and 100% specificity. Hohenthal et al. reported that among 135 BAL fluid samples from patients with hematologic malignancies diagnosed with pneumonia from 1996 to 2002, CMV PCR was positive in 18 (13.4%), 4 (22.2%) of which were obtained from patients with definite or probable CMV pneumonia. However, the significance of the positive PCR findings was unknown in 14 (77.8%) patients [24]. Since then, no study on the feasibility of CMV detection by PCR using BAL fluid from patients diagnosed with hematologic malignancies has been reported. In lung transplant recipients, Chemaly's *et al*. [17] had described the viral cut-off from BAL fluid as 500,000 copies/mL. This viral cut-off is quite different from ours; however, we assume that this discrepancy could have been driven by different host immune status and the much smaller study population in Chemaly's study. Furthermore, to our knowledge, no other study has evaluated use of a qPCR method to discriminate CMV pneumonia from CMV infection. Our study is unique in terms of its use of qRT-PCR to enable a quantitative comparison and inclusion of a considerably larger population than those of previous works.

BAL is the standard method used to detect viral pathogens of pneumonia in patients with hematologic malignancies [14]. In our BMT center, we perform bronchial washing after wedging the bronchoscope at one selective segmental bronchus to identify the pathogen (s) present in pulmonary infiltrates while minimizing alveolar damage. The incidence of CMV pneumonia in our study was well corresponded to previous reports [2, 24], with the simplified bronchial washing procedure than BAL.

This study had several limitations. First, 10 patients in whom CMV infection was not proven by lung biopsy or culture were included in the ROC curve analysis. This clinical diagnosis of CMV pneumonia is debatable, but an experienced specialist excluded patients with other etiologies of infection and non-infectious conditions from the analysis by means of a strict review. As a result, all patients were treated with anti-CMV agents and/or CMV immunoglobulin, and none improved spontaneously without antiviral agents. Also, the percentage of diagnosed specimens among the studied samples was 4.2%, in the range of the values reported by Cathoma (8.3%) [2] and Hohenthal (3.0%) [24]. Second, among the 24 diagnosed patients, 8 had not undergone BMT prior to diagnosis of CMV pneumonia. Because our center has been conducting risk-adapted pre-emptive therapy after BMT since 2000 according to BMT type and GVHD grade [12, 25, 26], four patients diagnosed with CMV pneumonia had histories of pre-emptive therapy after BMT. Since strategies to prevent CMV pneumonia and diagnosis in non-BMT patients with hematologic malignancies remain to be determined [3, 27–29], none of the eight non-BMT patients had histories of pre-emptive therapy for CMV disease. We separately analyzed the ROC curves of CMV titers with reference to BMT status. The viral cut-off was 18,900 copies/mL (137,970 IU/mL) in post-BMT patients and 316,415 copies/mL (2,309,825 IU/mL) in no-BMT patients. The viral cut-off of no-BMT patients was much higher than that of post-BMT patients; this difference is clinically relevant. Due to the long-term immune suppression required post-BMT and transfer of virus from seropositive donors, whether or not a patient underwent BMT influences the risk of CMV reactivation to end-organ disease. We found that exclusion of no-BMT patients made little difference to the determined cut-offs level, which decreased from 28,774 copies/mL (210,054 IU/mL) to 18,900 copies/mL (137,970 IU/mL). However, only 16 post-BMT patients were diagnosed with CMV pneumonia in present study; this small number may have compromised the accuracies of the ROC curves. CMV titers determined by qRT-PCR should be analyzed according to BMT status and risk of CMV disease in a larger population to obtain statistically significant data. Moreover, the cut-offs level should be validated prospectively derived from and at the same time applied to the same dataset. Third, pulmonary hemorrhage may be in play; the viral load in BAL may reflect CMV reactivation in blood. We identified 3 patients with pulmonary alveolar hemorrhage among the 24 diagnosed with CMV pneumonia, and 1 case of hemorrhage among the 70 patients not diagnosed with CMV pneumonia. In the ROC curve drawn after exclusion of patients who possibly had pulmonary hemorrhages, the viral cut-off value was 19.420 copies/mL (185,682 IU/mL) (AUC 0.923 ± 0.031, 95% CI 0.863-0.983; *P* < 0.001: sensitivity 83.8%, specificity 65.6%). Thus, the exclusion of patients with pulmonary hemorrhages created only minimal interval changes in the cut-off values, possibly because the blood CMV titers were low in such patients (data now shown). Lastly, we also reviewed the effect of antiviral treatment prior to bronchoscopy. Two such patients were identified; they had been pre-emptively treated with ganciclovir under suspicion of CMV reactivation upon examination of blood CMV PCR titers. However, the intervals between the commencement of the antiviral agent and bronchoscopy were relatively short, (11 and 5 days); any effect would be expected to be minimal.

## CONCLUSIONS

CMV loads in bronchial washing fluid and whole blood determined by qPCR could indicate lung involvement in CMV disease. A cut-off value of 28,774 copies/mL (210,054 IU/mL) CMV DNA in bronchial washing fluid was correlated with CMV pneumonia.

## SUPPLEMENTARY FIGURE



## Abbreviations

BMT: bone marrow transplantation
CMV: cytomegalovirus
CT: computed tomography
GVHD: graft-versus-host disease
IHC: immunohistochemical
PCR: polymerase chain reaction
qRT-PCR: quantitative real-time PCR
