# Supplementary material for: Diagnosis of cytomegalovirus pneumonia by quantitative polymerase chain reaction using bronchial washing fluid from patients with hematologic malignancies
Source: Oncotarget. 2017 Jan 4;8(24):39736–45. doi: 10.18632/oncotarget.14504 (PMC5503648; doi:10.18632/oncotarget.14504)
Supplement: Supplementary file 1 [file oncotarget-08-39736-s001.pdf]

Diagnosis of cytomegalovirus pneumonia by quantitative polymerase chain reaction using bronchial washing fluid from patients with hematologic malignancies

Hwa Young Lee[1,*], Chin Kook Rhee[1,*], Joon Young Choi[1], Hea Yon Lee[1], Jong Wook Lee[2,3] and Dong Gun Lee[3,4,5]

[1] Department of Internal medicine, Division of Pulmonary and Critical Care Medicine, The Catholic University of Korea, Seoul, Korea

[2] Department of Internal Medicine, Division of Hematology, The Catholic University of Korea, Seoul, Korea

[3] The Catholic Blood and Marrow Transplantation Center, The Catholic University of Korea, Seoul, Korea

[4] Department of Internal medicine, Division of infectious diseases, The Catholic University of Korea, Seoul, Korea

[5] Vaccine Bio Research Institute, The Catholic University of Korea, Seoul, Korea

[*] These authors have contributed equally to this work

***Correspondence to**: Dong Gun Lee, **email**: symonlee@catholic.ac.kr*

Keywords: cytomegalovirus, pneumonia, real-time polymerase chain reaction, hematologic neoplasms

***Received**: August 04, 2016* ***Accepted**: December 27, 2016* ***Published**: January 04, 2017*

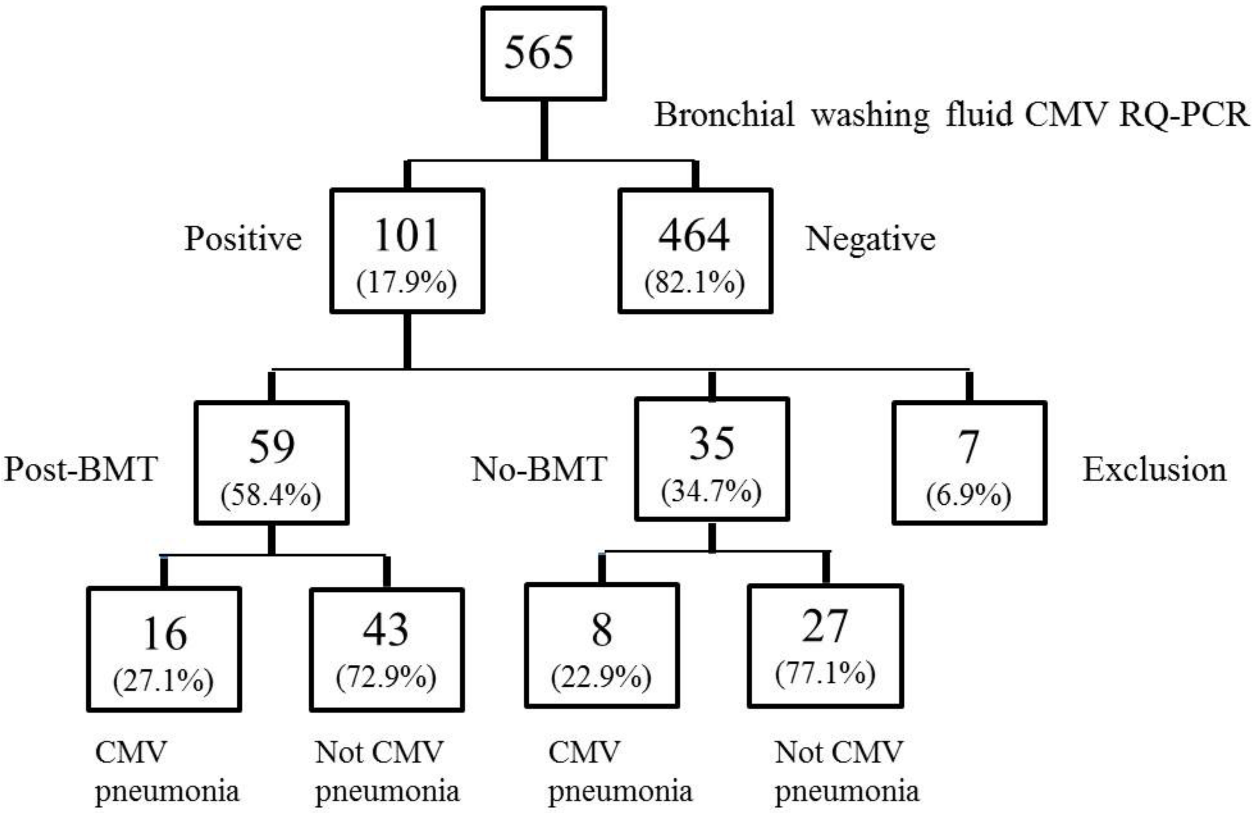

Figure S1: Flow chart of 101 patients who harbored > 380 copies/mL qRT-PCR results in bronchial washing fluid.
